# Measles in Ugandan Children Under 18 Months of Age: A Retrospective Study of Case-based Surveillance, 2018–2024

**DOI:** 10.1093/ofid/ofag459

**Published:** 2026-07-24

**Authors:** Gerald Bright Businge, Ezekiel Mupere, Samuel Ofori Gyasi, Kirsty Le Doare, Michael Boele van Hensbroek, Merijn W Bijlsma, Merryn Voysey

**Affiliations:** Makerere University-Johns Hopkins University (MU-JHU) Research Collaboration, Kampala, Uganda; Emma Children's Hospital, Amsterdam UMC, University of Amsterdam, Amsterdam, The Netherlands; Makerere University College of Health Sciences, School of Medicine, Kampala, Uganda; Uganda National Expanded Program for Immunisation, Ministry of Health, Kampala, Uganda; Makerere University-Johns Hopkins University (MU-JHU) Research Collaboration, Kampala, Uganda; City St. George's, University of London, London, UK; Emma Children's Hospital, Amsterdam UMC, University of Amsterdam, Amsterdam, The Netherlands; Emma Children's Hospital, Amsterdam UMC, University of Amsterdam, Amsterdam, The Netherlands; Oxford Vaccine Group, Department of Paediatrics, University of Oxford, Oxford, UK

**Keywords:** measles, measles surveillance, measles-rubella vaccine, vaccination

## Abstract

**Background:**

Measles remains a significant public health threat in Uganda, with regular outbreaks. The current national immunization schedule offers the first measles-containing vaccine dose (MCV) at 9 months, and a second dose at 18 months. We investigated the epidemiology of measles in Ugandan children aged 18 months and younger.

**Methods:**

We analyzed the national measles surveillance data from 2018 to the first quarter (Q1) of 2024, probing the demographic and geographic distribution. We categorized children as under 6 months, 6 to under 9 months, and 9–18 months. Suspected measles cases were classified as clinical, epidemiologically linked, laboratory-confirmed or discarded (not measles).

**Results:**

1316/2274 (57.9%) of the suspected cases were diagnosed with measles. One hundred and forty-nine cases (11.3%; 95% CI: 9.7–13.0) were aged under 6 months, 390 (29.6%; 27.0–32.0) 6 to under 9 months, and 777 (59.0%; 56.0–62.0) 9–18 months. 717/1208 (59.4%; 57.0–62.0) of vaccine-eligible children, with a captured vaccination history, had received at least 1 dose of MCV. 59.5% (95%CI: 55.0–63.0) of measles cases were unvaccinated vaccine-eligible children. Several districts did not achieve the WHO-recommended measles surveillance threshold of at least 2 discarded cases per 100 000 population.

**Conclusions:**

Most measles cases among Ugandan children ≤18 months are unvaccinated. Infants nine to 18 months old comprise most of the measles cases (59.0%; 777/1316). To achieve better control of measles transmission, review of the current vaccination policies with strengthened vaccination and surveillance systems are recommended.

Despite the availability of a safe and effective vaccine, measles continues to be a major cause of illness and death among young children worldwide [1, 2]. The introduction of the measles vaccine in the 1960s significantly reduced measles-related outbreaks and mortality globally [3]. However, measles remains prevalent in many low- and middle-income countries, particularly in Africa and Asia. These regions have suboptimal vaccination coverage (<95% receiving 2 doses) required to meet measles elimination efforts [4]. A joint CDC/WHO and partner report, using a modeling approach with surveillance data input from member countries for the year 2022, reported a global estimate of 9.2 million measles cases and over 136 000 deaths; two-thirds of these deaths occurring in the African region [4].

Measles outbreaks are frequent in Uganda, despite the country's reported coverage of 93% in 2023 for the first measles-containing vaccine dose [5]. Uganda aims for a ≥95% national vaccination coverage [6], with the first dose of the measles-containing vaccine (MCV1) given at 9 months of age, followed by a second dose at 18 months (MCV2) introduced in 2022. The latter dose introduction was per the World Health Organization (WHO) recommendation [7]. Two measles vaccine doses at 95% coverage are the required threshold to confer optimal individual and community protection (herd immunity) against measles infections [7, 8]. The 2023 WHO and UNICEF reports estimate Uganda's measles vaccination coverage, as of 2023, to be 90% and 21% for MCV1 and MCV2, respectively [9]. While the routine national vaccination schedule targets children at 9 months for MCV1 and 18 months for MCV2, infants 6 to under nine months are included in national or regional supplementary immunization activities (SIAs). Despite this routine vaccination and SIAs, the country continues to grapple with meeting the 2020 WHO AFRICA measles surveillance and elimination targets. The country's measles incidence rates have remained above one confirmed case per million population per year [10]. Between 2016 and 2020, the country's measles incidence was between 7 and 66 cases per million population, with a significantly higher incidence in children under 5 years at 451 cases per million compared with 65 cases per million in the rest of the population [10].

In utero transfer of maternal antibodies provides temporary protection against measles in early infancy, but these antibodies typically decline to nonprotective levels between 3 and 9 months of age [11, 12]. The rate of this decline varies depending on whether the mother acquired immunity through natural infection or vaccination, with protection obtained via the latter thought to wane faster in the infant [13–15]. This creates a potential window of vulnerability in infants before they receive their first measles-containing vaccine (MCV) dose at nine months. An epidemiological analysis of measles among young infants by the WHO and US CDC between 2011 and 2016 showed the highest age-specific annual measles incidence to be in children 6–8 months at about 213 cases per million, closely followed by children 9–11 months at 191 cases per million children [16]. Analysis of measles case-based surveillance data in Uganda between 2016 and 2020 revealed that the majority of cases (59%) were children aged under 5 years, followed by those aged 5–9 years (22%), 10–14 years (10%) while individuals aged 15 years and over made up 9% of the cases [17]. Outbreak related reports have described case fatalities of 5%–17% in children aged 4 months to 3 years [18, 19].

Vaccination of infants earlier, close this window of vulnerability, has historically been contested because maternal antibodies may modulate the immune response following vaccination. It is postulated that the young infants’ immune system, particularly the humoral system, may not be sufficiently developed at the time of an earlier vaccination to enable a robust immune response [20–22].

This study aimed to investigate the incidence and prevalence of measles disease in Ugandan children aged 18 months and younger. At the age of 18 months, every child is expected to have received at least 1 dose of the measles-containing vaccine (MCV).

## METHODS

### Study Design and Data Collection

This study utilized anonymized surveillance data that is routinely submitted to the Uganda National Expanded Program on Immunization (UNEPI) at the Ministry of Health (MoH), Uganda. Measles is one of the priority diseases for immediate reporting under the Ugandan Integrated Disease Surveillance and Response (IDSR) [23]. The IDSR captures information on notifiable illnesses via a 2-pronged approach; the indicator-based surveillance (IBS) and event-based surveillance (EBS). The IBS involves systematic identification, collection, reporting, and monitoring of health-based indicators such as a “febrile rash illness” with cough or conjunctivitis or coryza (“Measles-like illness,” MLI) for measles disease, while EBS is anchored on investigating signals at the community level that may indicate a potential disease outbreak. Weekly, monthly, quarterly, and annual reports of conditions managed at health facilities are reviewed, and suspicious cases are flagged. The IBS also includes a case-based surveillance component requiring active laboratory testing for measles (immunoglobulin M [IgM]), plus follow-up of identified cases. Reported suspected measles cases are registered and evaluated at the national Expanded Program on Immunization (EPI) laboratory at the Uganda Virus Research Institute [23, 24].

### Definitions

We defined our surveillance cohort as all the reported suspected measles cases aged 18 months or younger. Measles-containing vaccine dose vaccine-eligible children were defined as children in the surveillance cohort aged 9 months or older.

The final classification of suspected measles cases was per the WHO definitions [25] whereby (1) a (laboratory) confirmed case was a suspected case in which measles was confirmed by an accredited laboratory, (2) an epidemiologically linked (Epi-linked) case lacked laboratory investigations/confirmation but was geographically and temporally related to a confirmed or other Epi-linked measles case, (3) a clinically compatible case was one presenting with measles-like symptoms, without an adequate clinical specimen collected, and not epidemiologically linked to a laboratory-confirmed case of measles or other communicable disease, and (4) a discarded case was a sufficiently investigated measles case and adjudicated to be a nonmeasles case.

Measles cases in this study were children in the surveillance cohort classified as either clinically compatible, Epi-linked, or laboratory-confirmed diagnoses of measles.

### Data Analysis

Anonymized data were abstracted from the national surveillance database as a Microsoft Excel file. In consultation with the statistical lead in the monitoring and evaluation department at UNEPI, missing or unknown data parameters were defined. Records with completely missing or unknown data points were excluded from this analysis.

We obtained data on the final measles classification, age, sex, vaccination status, and the reporting district. We further obtained the national and district-specific infant population projections from 2015 through 2024 from the national statistics office's website—the Uganda Bureau of Statistics [26]—to compute age-specific incidence per district.

We summarize continuous variables as median with interquartile range and categorical variables using proportions. Categorical variables were compared using the Chi-square test, with a *P* value of <.05 considered for statistical significance. The surveillance cohort was categorized into children under 6 months, 6–<9 months, and 9 to 18 months to accommodate age groups of vaccination during routine or SIA vaccination activities. We assessed the measles prevalence by age groups, the national incidence of measles in this pediatric population at district level, as well as district surveillance performance. The WHO criteria for measles surveillance performance of at least 2 discarded cases per 100 000 population per 12 months was applied. This is considered the effective threshold in achieving measles elimination and describing residual measles virus transmission or elimination [27].

All analyses and spatial illustrations in this study were performed using R statistical software (version 4.4.2).

### Ethical Consideration

The data used for this study were anonymized. Access to the anonymized data used for this study was granted through the office of the Director General, Ministry of Health, Uganda. Data access and processing were done in accordance with the Uganda Health Data Access, Sharing and Use Guidelines [28] and the Data Protection and Privacy Act 2019 [29]. All requests for use and publication of results from this review were subject to administrative review to ensure protection of health-related information and compliance with the Ugandan law.

## RESULTS

During this 7-year period, 2274 suspected measles cases were reported in children aged 18 months and younger. Three children did not have a final classification at the time of data extraction. Over half of the suspected cases, 1316/2271 (57.9%) were classified as measles cases (ie, a clinical, Epi-linked or laboratory-confirmed measles case) while 955/2271 (42.1%) were discarded. The highest number of confirmed cases was in 2019, with 201/433 (46.4%) of the suspected cases being laboratory-confirmed measles cases. Nearly all suspected cases in 2021, 132/139 (95.0%), were discarded (Table 1).

**Table 1.** Number of Suspected Measles Cases in Children Under 18 Months by Final Classification

Final Classification	2018	2019	2020	2021	2022	2023	2024 (Q1)	Total
	n (% of all)	n (% of all)	n (% of all)	n (% of all)	n (% of all)	n (% of all)	n (% of all)	N (%)
	(% of tested)^a^	(% of tested)	(% of tested)	(% of tested)	(% of tested)	(% of tested)	(% of tested)	
Clinical^b^	319 (48.9)	12 (2.8)	1 (0.5)	2 (1.4)	38 (21.3)	0 (0.0)	0 (0.0)	372 (16.4)
EPI-linked^c^	54 (8.3)	95 (21.9)	83 (39.3)	0 (0.0)	1 (0.6)	87 (19.7)	0 (0.0)	320 (14.1)
Confirmed^d^	183 (28.1)	201 (46.4)	66 (31.2)	4 (2.9)	23 (12.9)	71 (16.1)	76 (34.7)	624 (27.4)
	(65.6)	(61.7)	(52.0)	(2.9)	(16.5)	(20.1)	(34.7)	
Discarded^e^	96 (14.7)	125 (28.9)	61 (28.9)	132 (95.0)	116 (65.2)	282 (63.8)	143 (65.3)	955 (42.0)
	(34.4)	(38.3)	(48.0)	(97.1)	(83.5)	(79.9)	(65.3)	
Pending^f^	0 (0.0)	0 (0.0)	0 (0.0)	1 (0.7)	0 (0.0)	2 (0.5)	0 (0.0)	3 (0.1)
Laboratory tested (all)	279 (42.8)	326 (75.3)	127 (60.2)	136 (98.6)	139 (78.1)	353 (80.2)	219 (100.0)	1579 (69.5)
Total	652	433	211	139	178	442	219	2274

^a^Tested: Children with a laboratory test done that is “confirmed” and “discarded” cases only.

^b^Clinical: suspected measles case without an adequate clinical specimen collected for laboratory investigation or EPI-linked.

^c^Epi-linked: suspected measles case without a laboratory confirmation, however, geographically and temporally related to a confirmed or Epi-linked case.

^d^Confirmed: Suspected measles case that is laboratory confirmed to be measles.

^e^Discarded: Sufficiently investigated measles case and determined to be a nonmeasles and nonrubella case.

^f^Pending: pending a final measles classification.

### Baseline Characteristics of the Surveillance Cohort

The median age of the surveillance cohort was 10 months (interquartile range 7, 14 months), with nearly a third of the children aged under nine months (35.5%). There was a slight male predominance (57.0%) in this cohort. Nearly two-thirds (717/1208, 59.4%) of MCV vaccine-eligible children, with a known vaccination history, had received at least 1 MCV dose (Table 2). Characteristics of the measles cases in this surveillance cohort are provided in Supplementary Table 1.

**Table 2.** Characteristics of Children 18 Months and Younger Reported in the Measles Surveillance 2018 Through the First Quarter of 2024

Characteristic	Median, IQR	All Children, N = 2274 (%)
Gender (N = 2273)		
Male		1296 (57.0)
Female		977 (43.0)
Age in months	10 (7,14)	
Age category		
Under 6 m		232 (10.2)
6 to under 9 m		575 (25.3)
9 m to 18 m		1467 (64.5)
Vaccination status (N = 1467)^a^		
Vaccinated		717 (48.9)
Unvaccinated		491 (33.5)
Unknown		259 (17.7)
Measles Classification (N = 2271)		
Confirmed		624 (27.5)
Clinical		372 (16.4)
Epidemiologically linked		320 (14.1)
Discarded		955 (42.1)

^a^MCV vaccine-eligible children (9–18 m of age).

**Figure 1.**
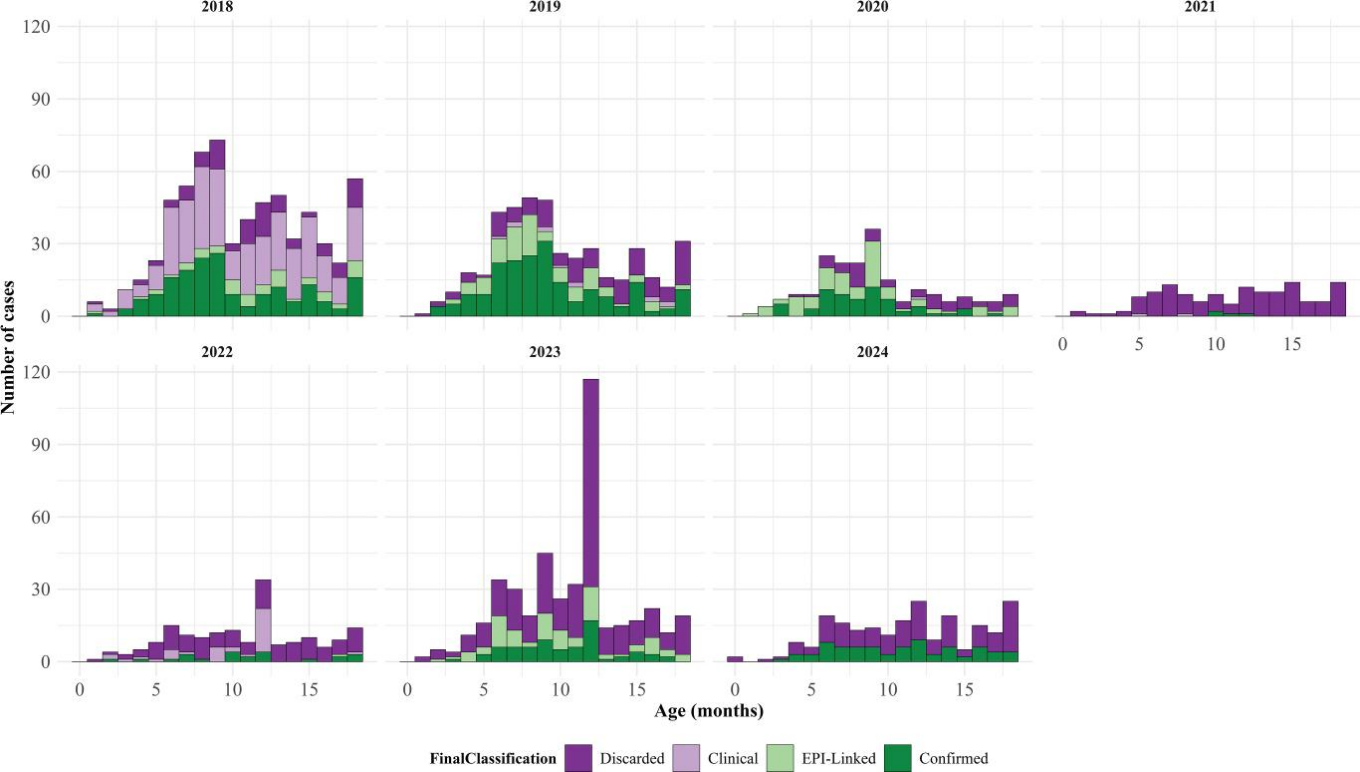
Distribution of measles cases in Ugandan children under 18 m: 2018 to Q1 2024.

Between 2020 and 2022, there was a progressive decline in the number of suspected measles cases registered, with the lowest reports in 2021. This coincided with the Coronavirus 19 disease (COVID-19) pandemic. However, in 2023 and quarter one of 2024, the number of suspected cases seemed to gradually return to the prepandemic numbers. We observed a peak age of 9 months among the reported suspected cases in both the prepandemic and pandemic period, and about 12 months in the postpandemic period (Figure 1).

Children under the routine age of MCV1, at nine months, accounted for more than a third of the measles cases, 539/1316 (41.0%, 95% CI: 38.0–44.0). The majority of these (390/539, 72.4%) were aged 6 to under 9 months of age. Measles virus infection was laboratory confirmed in 267/539 (49.5%) in this same age group, suggesting insufficient measles protection before nine months, particularly in the 6 to nine months age window (Table 3).

**Table 3.** Final Measles Distribution and Vaccination Status of Surveillance Cases by Age

Characteristic		Age Group		*P* Value
	<6 m	6–<9 m	9–18 m	
	N = 232 (%)	N = 575 (%)	N = 1467 (%)	
Vaccination status				<.001
Vaccinated	0 (0.0)	67 (11.7)	717 (48.9)	
Unvaccinated	149 (64.2)	393 (68.3)	491 (33.5)	
Unknown	83 (35.8)	115 (20.0)	259 (17.7)	
Classification				<.001
Laboratory confirmed	68 (29.3)	199 (34.6)	357 (24.4)	
Clinical	34 (14.7)	97 (16.9)	241 (16.5)	
Epidemiologically linked	47 (20.3)	94 (16.4)	179 (12.2)	
Discarded	83 (35.8)	185 (32.2)	687 (46.9)	
Measles illness (n = 2271)				<.001
Yes	149 (64.2)	390 (67.8)	777 (53.1)	
No	83 (35.8)	185 (32.2)	687 (46.9)	

	Measles classification^a^		
	Measles	Not Measles	
	N = 777 (%)	N = 687 (%)	
Vaccination status			<.001
Vaccinated	251 (32.3)	464 (67.5)	
Unvaccinated	368 (47.4)	122 (17.8)	
Not known	158 (20.3)	101 (14.7)	
Classification			<.001
Confirmed	357 (45.9)	-	
Clinical	241 (31.0)	-	
Epidemiologically linked	179 (23.0)	-	
Discarded	-	687 (100.0)	

^a^Measles classification among vaccine-eligible children (9–18 m), with a known final measles classification.

Among vaccine-eligible children with a recorded vaccination history, 368/619 (59.5%, 95% CI: 55.0–63.0) were unvaccinated. Collectively for the entire surveillance cohort, 765/1046 (73.1%, 95%CI: 70–76) of the measles cases were unvaccinated children (Supplementary Table 1). Sixty-seven children (11.7%) aged 6 to under 9 months had a documented vaccination history, likely due to supplementary immunization activities in 2019 and 2022. Nearly half, 717/1464 (48.9%), of vaccine-eligible children had received at least 1 dose of the vaccine, while no child under 6 months had received measles vaccination (Table 3). The vaccination status and final measles classification of this surveillance cohort versus vaccine-eligible children is provided in Supplementary Table 2.

**Figure 2.**
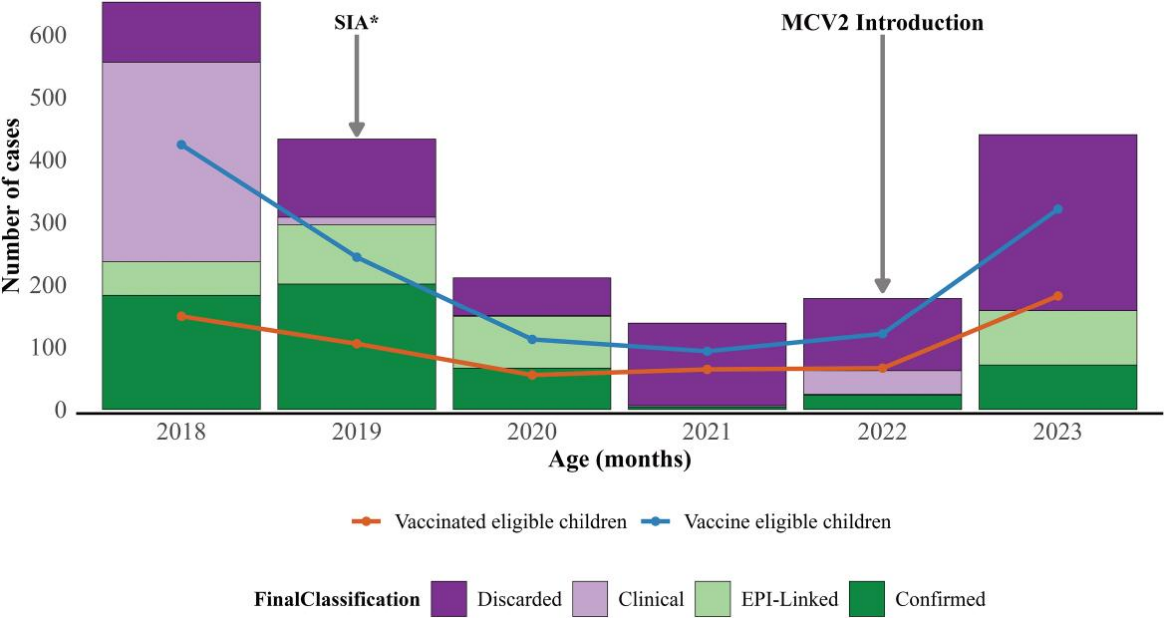
Proportion of MCV-eligible children and measles cases per year: 2018–2023.^1,2,3^. * Supplemental immunization activities (SIA). ^1^The SIA in October 2019 involved the vaccination of children 6 m to 15 y of age at 1 or more locations within a short interval of time.^2^ The second measle containing vaccine dose (MCV2) was introduced in the routine immunization program in 2022.^3^ Trends assessed for years with a complete 12-m reporting (2018 through 2023).

The proportion of vaccinated MCV-eligible children in this surveillance cohort varied throughout the observation period. The lowest proportion of vaccination was 35.1% in 2018 and the highest was 68.8% in 2021. Overall, an inverse relationship between the number of measles cases and the proportion of children vaccinated during this study period (Figure 2).

The highest proportion of discarded cases was in children aged 9–18 months (687/1464 (46.9%)) versus 185/575 (32.2%) and 83/232 (35.8%) in the 6 to under nine months and less than 6 months age groups, respectively.

### Geographical Distribution of Measles Cases

**Figure 3.**
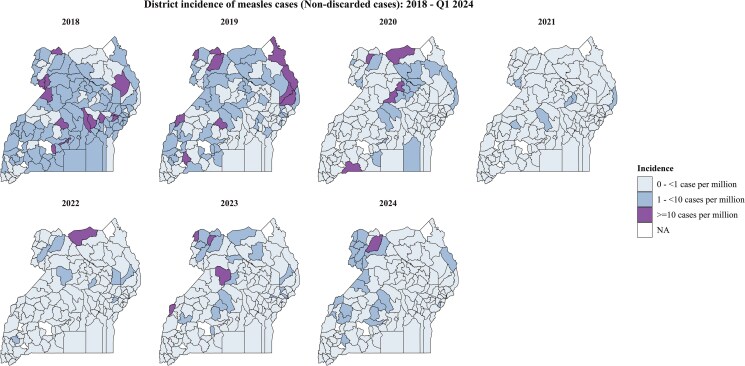
Total measles incidence per million in children aged under 12 m. Rates computed for the under-1-year child population per the available national under-1-year population estimates^1^. ^1^NA—Data not available for the district.

**Figure 4.**
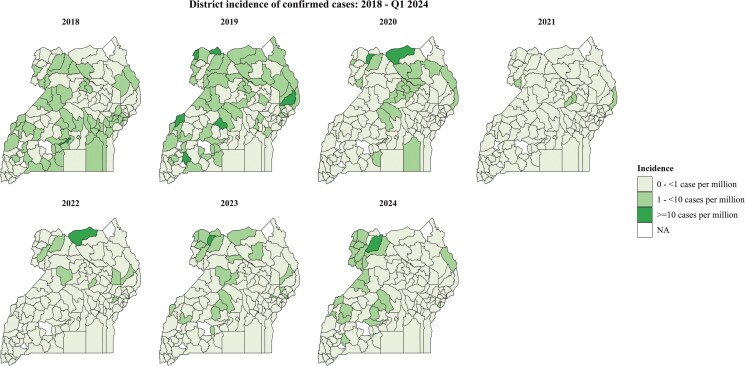
Confirmed measles incidence per million in children under 12 m. Rates computed for the under-1-year child population per the available national <1 y population estimates. ^1^NA—Data not available for the district.

**Figure 5.**
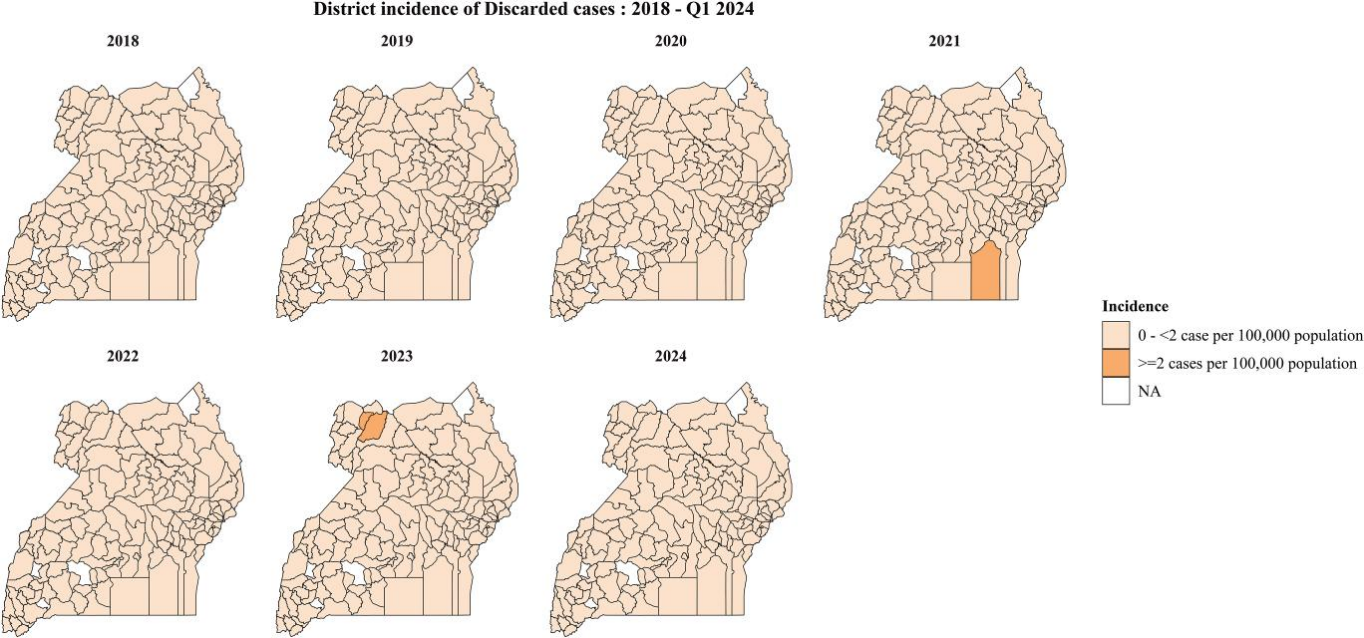
Discarded cases per 100 000 population. Rates computed for the under-1-year child population per the available national <1 y population estimates. ^1^NA—Data not available for the district.

**Figure 6.**
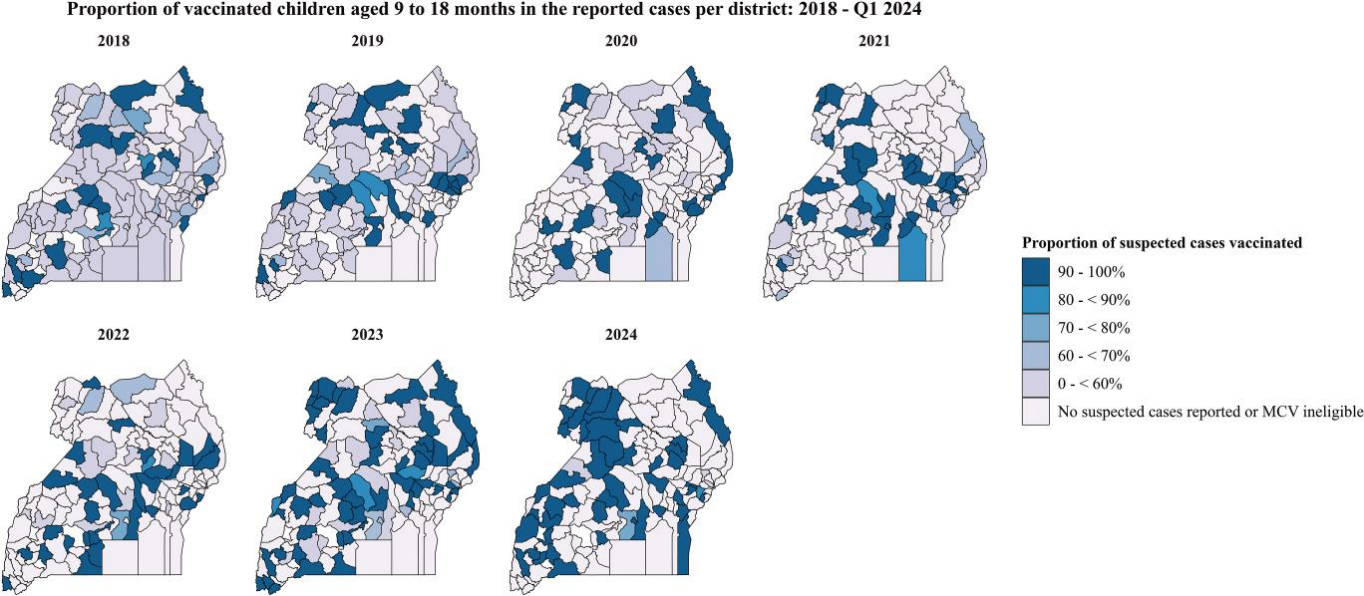
Proportion of vaccinated measles containing vaccine (MCV) eligible children among reported suspected cases per district.

For this observation period, at least 1 suspected measles case was reported from each district, where data were available. The highest geographical clustering of measles cases was in the central, southern, and border districts (Figures 3 and 4). This geographical pattern was relatively consistent throughout the review period. However, differences were noted in the number of discarded cases reported from each district. Throughout the 7-year observation period, 44 districts attained the WHO-recommended surveillance threshold of at least 2 discarded cases per 100 000 population (Figure 5). We also observed a heterogeneous representation of MCV-eligible vaccinated children during this period, with higher vaccination rates in the central, northwestern and southwestern districts having received MCV (Figure 6).

## DISCUSSION

Our study demonstrates a significant burden of measles disease in Ugandan children under 18 months, particularly children aged 6 to under 9 months. There was a general reduction in the number of measles cases toward and including 2020, when the COVID-19 pandemic lockdown was instituted, with a resurgence to the prepandemic rates after this period. However, the number of reported suspected measles cases might be underrepresented as several districts in the country did not meet the recommended measles surveillance threshold.

Uganda subscribes to the WHO recommendation for vaccinating infants at 9 months in high-burden settings [7]. This guidance hinges on the expected persistence of protective maternal antibodies in the infant before requiring measles vaccination. However, decay of maternal antibodies below the threshold of protection as early as 3–4 months has been reported [12]. In this study, infants aged 6–9 months accounted for over two-thirds (390/539, 72.4%) of measles cases in children below 9 months. This is corroborated by an epidemiological investigation following a measles outbreak in Kakumiro district by Okiror and colleagues. The authors reported an attack rate 30% higher in younger children to the older peers (9 months to <5 years) [30]. Biribawa et al [18] in their evaluation of a measles outbreak in Lyantonde and bordering districts, showed the attack rate in children under 9 months to be nearly twice as high as that in the general population (17/100 000 vs 9/100 000). Their study, however, had small participant numbers and purposively enrolled more children aged 1 year or older. Epidemiological studies by Polycarp among Nigerian children revealed similar observations, with disproportionately high measles incidence in younger infants [31]. These studies demonstrate the significant risk of measles disease that infants aged 6 to under 9 months have.

Measles disease tends to be more severe in younger children [19, 32, 33], with the incidence of severe disease several-fold higher than that of the general population [10, 34]. With over two-thirds of the measles cases in our study being children aged 6 to under 9 months, there is a potential argument for reviewing the timing of the first MCV. A meta-analysis by Ong and colleagues on the seroprevalence of maternal measles antibodies among infants aged 9 months or younger in low- and middle-income countries revealed up to 70% of the infants were seronegative by the age of 4 months [35]. The findings from this study and the previous works underscore the ongoing vulnerability and need to offer protection in this age group. This would align with the growing body of knowledge on the potential protective role an earlier vaccination schedule may confer before the current 9-month schedule [36–38]. We would recommend such considerations be balanced against concerns that the MCV administered before 9 months of age may be less effective [39]. Failed seroconversion postmeasles vaccination, however, is more pronounced under 6 months of age, likely due to circulating neutralizing maternal antibodies [40] and an immature infant immune system [39, 41]. Further studies are needed to establish the optimal change to the vaccination schedules.

We noted nearly two-thirds of measles cases in vaccine-eligible children (≥9 months) in our surveillance cohort were unvaccinated. The overrepresentation of unvaccinated individuals in measles cases has been observed in several other outbreak settings. Over 90% of measles cases were unvaccinated in a Somali study by Siad et al [42], while a 5-year review of measles trends in Tanzania revealed about 58% of laboratory-confirmed cases were unvaccinated [43]. However, the occurrence of measles in vaccinated individuals is also known to occur. These are usually breakthrough infections following non or poor response to measles vaccination. In our study, 251/619 vaccine-eligible children (40.5%, 95% CI 37.0–45.0) had a vaccination history recorded. While 1 dose of the measles vaccine is 85%–93% effective, full protection is achieved after 2 MCV doses [44, 45]. The second measles vaccine dose aims to protect individuals who might not have responded to the first dose. The overrepresentation of unvaccinated vaccine-eligible children in our study suggests a gap in the current measles control strategies or suboptimal uptake of vaccination services. World Health Organization estimates reported MCV1 coverage in Uganda at 90% as of 2023, while MCV2, newly introduced in the national immunization schedule in 2022, was at 21% [9]. However, there is a need to monitor this gap as dropouts at subsequent vaccination schedules beyond the WHO threshold of 10% have been described across several African countries [46]. Lowering the age of the first measles vaccination dose might have the potential to capture more children at risk of dropping out at subsequent vaccination encounters. It would also provide an earlier opportunity for parents/caretakers to be reminded about the second measles-containing vaccine dose. This approach would, however, need to be complemented by addressing the logistical and access barriers (such as distance to facilities, vaccine stock-outs, understaffed facilities), traditional and religious beliefs, and misinformation that frustrate optimal vaccine uptake across several regions in Uganda [47–50]. We recommend a review of the current public engagement strategies by the vaccination implementing and administrative stakeholders, such as the Ministry of Health, to address these shortfalls.

The COVID-19 pandemic disrupted several global and national public health infrastructures, with setbacks in immunization and surveillance programs [51]. The institution of a hard lockdown in March 2020 in Uganda negatively affected routine immunization services, which dropped by 19% within 3 months [51]. While the lockdown and other COVID-19 transmission mitigation measures, such as the use of face masks in public spaces, social distancing, and routine handwashing, may have reduced measles transmission in communities, the recalibration of surveillance systems to COVID-19, equally, might have resulted in fewer measles cases visiting health facilities or going unreported. This might explain the drop in numbers in the years 2020 through early 2022, where there was an overall declining trend of cases during this COVID-19 pandemic period, with progressive resurgence in cases thereafter. This was particularly prominent in the central, southern, and border districts.

We noted that most of the measles cases in our study were reported in the central, southern, and border districts. The central clustering could be explained by the high population in these regions. The country's capital city, located in the central district, is one of the largest business hubs. The large population in the central districts provide opportunities for community transmission of measles. The southern and border districts are home to several business hubs; however, these districts routinely provide refuge to nationals from neighboring countries such as South Sudan, Rwanda, the Democratic Republic of Congo and Somalia. Measles outbreaks originating and propagating from refugee settlements have been reported in Uganda and other regions [52, 53].

In our study, over 40% of suspected cases in the surveillance cohort were discarded. Unfortunately, the data available to the authors of this study did not provide the alternative diagnoses for the discarded cases as extended pathogen testing in discarded cases is not routine laboratory practice. However, works by Prossy et al [54] on other causes of measles-like illness identified viruses such as rubella, parvovirus B19, adenovirus and Epstein-Barr Virus as the causes of the rash-associated illness. The majority (58%) of these viruses were identified from samples collected in children under 5. Wang and colleagues found similar causal agents in this age group among Australian children [55]. In the absence of the classical dermatological features and laboratory testing, the symptomatic case definition of measles has a low specificity, especially in younger children. The use of a clinical case definitions to confirm suspected measles cases has been shown has been shown to have a sensitivity of 76%–88%, with a positive predictive value which inherently suffers as the measles incidence in the community falls [56]. With most districts in Uganda not meeting the WHO measles surveillance threshold, and the nonspecificity of symptoms used in the case definition, there is a need to improve or decentralize laboratory support for measles diagnosis in the country.

### Strengths and Limitations

This study has a balance of strengths and limitations. The 7-year dataset offers a robust longitudinal perspective on measles disease trends among children under 18 months of age. We were able to obtain valuable insights into this critically vulnerable population. By focusing on this age group, the study sheds light on the immunization coverage and disease dynamics in an essential and particularly vulnerable age group.

However, the study faced inherent challenges associated with the current surveillance system that included incomplete or missing parameters, such as the vaccination status and imprecise or missing age data. As we demonstrated, only 30% (44/147) of the reporting districts met the WHO threshold of surveillance. The burden of measles in this group might therefore be under reported in the data available in the surveillance system. These limitations were likely compounded by disruptions following the COVID-19 pandemic. The study was also constrained in its ability to generate incidence rates specifically for children aged 18 months across the surveillance years. This was due to the lack of national population estimates for this precise age group, which are typically aggregated into broader categories, such as under 12 months, making detailed age-specific analysis challenging. However, working with the statistician at the Uganda EPI team, the team was able to obtain clarification for the unclear nonmissing data descriptions. The authors also restricted the computation of the measles incidence rate to the available population data for infants at the district and national level—children ≤1 year. The HIV-1 (HIV) status of mothers and/or the infants was not available to the authors to evaluate possible associations been HIV status and measles status, especially among the younger children. Previous studies in Kenya, Malawi and India have reported reduced maternal measles transfer ratios in HIV exposed infants and a 2–4 fold higher risk of measles in the same group [57–60]. We also acknowledge the potential role of SIAs at interrupting ongoing community transmission of measles and consequently, a reduction in number of registered suspected measles cases. This was demonstrated by a drop in measles positivity rates from 41.88% (95% CI: 39.30–44.51) to 37.96% (95% CI: 32.81–43.40) among surveillance cases following the 2019 SIA [61].

### Implications of Results

The number of measles cases among infants aged 6 to under 9 months underscores an unmet need to reassess the timing of the first measles-containing vaccine dose. An earlier administration of the measles-containing vaccine could potentially reduce the disease burden in this vulnerable age group. However, it remains crucial to further study and address the concern about the effectiveness of the vaccine when given at a younger age. Interventions that balance these factors are essential to protect the most susceptible populations and mitigate the spread of measles. Exploration of optimal vaccination schedules, in tandem with community advocacy to improve uptake of vaccination services, can ensure that revised vaccination strategies are both effective and properly tailored to the needs of these vulnerable young members of our communities.

### Conclusion

This study's findings demonstrate vaccination coverage and timing as pivotal to the mechanisms required to interrupt measles transmission. With most measles cases in our study being unvaccinated children, particularly infants aged 6 to under 9 months, an argument to review the current vaccination schedules in Uganda and other high-burden countries in general is raised. Evidence to support the feasibility and potential efficacy of introducing earlier measles vaccination doses is still required, nonetheless.

## Supplementary Material

ofag459_Supplementary_Data

## Supplementary Data

Supplementary materials are available at *Open Forum Infectious Diseases* online. Consisting of data provided by the authors to benefit the reader, the posted materials are not copyedited and are the sole responsibility of the authors, so questions or comments should be addressed to the corresponding author.
