# Supplementary material for: Measles in Ugandan Children Under 18 Months of Age: A Retrospective Study of Case-based Surveillance, 2018–2024
Source: Open Forum Infect Dis. 2026 Jul 24;13(8):ofag459. doi: 10.1093/ofid/ofag459 (PMC13430662; doi:10.1093/ofid/ofag459)
Supplement: ofag459_Supplementary_Data [file ofag459_supplementary_data.docx]

**Measles in Ugandan Children Under 18 Months of Age: A retrospective study of case-based surveillance, 2018 – 2024**

# **Supplementary Tables**

# Supplementary Table 1: Baseline characteristics of measles cases

| **Characteristic** | **Median (IQR)** | **All measles cases (%) 1,316**^1^ |  |
| --- | --- | --- | --- |
| **Age** | 9 (7, 13) |  |  |
| **Sex** |  |  |  |
| Female |  | 587 (44·6%) |  |
| Male |  | 729 (55·4%) |  |
| **Final measles classification** |  |  |  |
| Confirmed |  | 624 (47·4%) |  |
| Clinical |  | 372 (28·3%) |  |
| Epidemiologically Linked |  | 320 (24·3%) |  |
| **Age category** |  |  |  |
| < 6 months |  | 149 (11·3%) |  |
| 6 - < 9 months |  | 390 (29·6%) |  |
| 9 - 18 months |  | 777 (59·0%) |  |
| **Vaccination status^‡^** |  |  |  |
| Vaccinated |  | 251 (32·3%) |  |
| Not Vaccinated |  | 368 (47·4%) |  |
| Not known |  | 158 (20·3%) |  |
| ^1^ n (%); Median (Q1, Q3)  ^‡^ Vaccine eligible children (≥ 9 months) | | | |

# Supplementary Table 2: Measles cases in all children (≤18 months) and vaccine-eligible infants in the study

|  | **All Children** | | | **Measles vaccine eligible children (9 - ≤18 months)** | | |
| --- | --- | --- | --- | --- | --- | --- |
|  | **Measles N = 1,316^1^** | **Not Measles N = 955^1^** | **p-value^2^** | **Measles N = 777^1^** | **Not Measles N = 687^1^** | **p-value^2^** |
| **Vaccination status** |  |  | <0·001 |  |  | <0·001 |
| Vaccinated | 281 (21·4%) | 501 (52·5%) |  | 251 (32·3%) | 464 (67·5%) |  |
| Not Vaccinated | 765 (58·1%) | 267 (28·0%) |  | 368 (47·4%) | 122 (17·8%) |  |
| Not known | 270 (20·5%) | 187 (19·6%) |  | 158 (20·3%) | 101 (14·7%) |  |
| **Final Classification** |  |  |  |  |  |  |
| Confirmed | 624 (47·4%) | 0 (0·0%) |  | 357 (45·9%) | 0 (0·0%) |  |
| Clinical | 372 (28·3%) | 0 (0·0%) |  | 241 (31·0%) | 0 (0·0%) |  |
| Epidemiologically Linked | 320 (24·3%) | 0 (0·0%) |  | 179 (23·0%) | 0 (0·0%) |  |
| Discarded | 0 (0·0%) | 955 (100·0%) |  | 0 (0·0%) | 687 (100·0%) |  |
| ^1^ n (%)  ^2^ Pearson’s Chi-squared test | | | | | | |
